# Choline supplementation in preterm infants: effects of four different supplements on choline plasma concentrations

**DOI:** 10.1007/s00394-025-03865-w

**Published:** 2026-01-12

**Authors:** Katrin A. Böckmann, Wolfgang Bernhard, Michaela Minarski, Anna Shunova, Julian Schwarz, Christian F. Poets, Axel R. Franz

**Affiliations:** 1 Department of Neonatology, Eberhard Karls University, Tübingen, Germany; 2 Center for Pediatric Clinical Studies, Eberhard Karls University, Tübingen, Germany

**Keywords:** Choline, Supplementation, Preterm infant

## Abstract

**Purpose:**

Current nutritional practices for preterm infants result in lower choline plasma concentrations than in a fetus matched for postmenstrual age. Choline is needed for growth and metabolism by membrane formation and plasma transport of polyunsaturated fatty acids (PUFA) via phosphatidylcholine (PC), and for methylation processes via betaine. Especially high concentrations of PUFA-PC are present in brain, eye, liver and lung; therefore, choline deficiency may contribute to developmental disorders of these organs. We studied short-term effects of four enterally administered choline compounds on plasma concentrations of choline-related parameters in preterm infants.

**Methods:**

Prospective study (June 2022–February 2024) in 32 enterally fed preterm infants (28.0–32.0 weeks gestation). Participants were randomized to receive an additional 30 mg/kg/d choline-equivalent in 6–8 doses with their meals for 48 h. Supplements: choline chloride, choline bitartrate, alpha-glycerophosphorylcholine (GPC) or egg-PC (of which GPC and PC are natural components of human milk). Blood was taken before meals < 72 h before start, 3 h or 6 h after start, and 3 h or 6 h after end of supplementation. Plasma concentrations of choline, betaine and PC were quantified by tandem mass-spectrometry.

**Results:**

Choline plasma concentrations and areas-under-the-curve (0–54 h) were similar between supplements. GPC increased choline concentration most rapidly (6 h, *p* = 0.01), and all supplements increased choline plasma concentrations at 51–54 h after start of supplementation, compared to baseline. There were no differential effects on betaine or PC plasma concentrations.

**Conclusion:**

Choline chloride, choline bitartrate, GPC and egg-PC increase choline plasma concentrations after 48 h of supplementation to a similar extent and are similarly suited for long-term choline supplementation.

**Study registration:**

This study was registered at the Deutsches Register Klinischer Studien (DRKS) (German Register for Clinical Studies), DRKS00020502.

**Supplementary Information:**

The online version contains supplementary material available at 10.1007/s00394-025-03865-w.

## Introduction

Choline is needed in every cell for the synthesis of phosphatidylcholine (PC) and sphingomyelin (SPH) for membrane formation. Moreover, PC is a major component of the secretions of the liver (bile, lipoproteins) and lung (surfactant) [1]. PC is the primary transporter of long-chain poly-unsaturated fatty acids (LC-PUFA), namely docosahexaenoic acid (DHA) and arachidonic acid (ARA) in plasma [2]. Further, choline is essential for the synthesis of acetylcholine as a neurotransmitter and for brain development [3]. Finally, and quantitatively important, choline’s oxidation product betaine is important for kidney function and as a methyl group donor for all methylation processes [4].

The fetus is supplied via the placenta, which actively transports choline into the fetus, achieving free choline plasma concentrations of approximately 40 µmol/L throughout the third trimester, i.e. threefold higher than in the pregnant mother [5]. After preterm birth, plasma levels decrease by 50%, or even more, within 48 h, rather than decreasing more gradually as in term born infants [5]. Preterm cessation of placental supply affects preterm infants during a phase of rapid growth and development, and reduced plasma levels in the preterm infant [5] may indicate that the (variable) choline contents of current formula and breastmilk [6, 7] may be too low for these infants, resulting in a cumulating choline deficit [8, 9].

Choline plasma concentrations define choline uptake into cells and parenchymal PC synthesis [10, 11]. During choline deficiency, choline is accreted by the liver from other organs [12]. Plasma choline concentrations below physiologic values are common in cystic fibrosis patients with exocrine pancreatic insufficiency [13, 14], and, in cystic fibrosis patients with decreased choline plasma concentrations, choline supplementation corrected plasma concentrations, increased precursor pools for PC synthesis and improved impaired function of both liver and lung [15]. Because preterm infants also have plasma choline concentrations below physiologic values between preterm birth and term equivalent age [5], and choline supplementation restored plasma concentrations to near-fetal levels [16], we postulated that choline supplementation may improve parenchymal choline homeostasis and clinical outcomes in preterm infants, too [5, 12, 15]. 

We have previously shown that enteral supplementation of an additional 30 mg/kg/d of choline as choline chloride, resulting in total enteral intakes of 50–60 mg/kg/day, nearly restores fetal plasma choline concentrations (36 [28–41] µmol/L vs. 19 [11–22] µmol/L) after 8-10d of supplementation [16], indicating that enteral choline supplementation is feasible. Moreover, we previously demonstrated that the plasma concentration time course and maximum increase from baseline and intestinal degradation to tri-methyl-amine (TMA) by bacteria with subsequent hepatic oxidation to TMA-oxide (TMAO) are different for individual choline components in adults [17, 18].

To prepare for long-term enteral choline supplementation in preterm infants (intending to maintain choline plasma concentrations of 35–40 µmol/l (like in utero) from early after birth to term equivalent age (i.e., 37–40 weeks postmenstrual age)), the aim of this study was to compare the effects of physiological choline components of breast milk (alpha-glycerophosphocholine [GPC] and PC), with the water-soluble standard compounds frequently used in infant formula (choline chloride and choline bitartrate), on water- and lipid-soluble choline derivatives in plasma. Additionally, we aimed to study changes in plasma concentrations of choline and its derivatives from baseline and their time course. Finally, TMAO was measured as an indicator of intestinal choline degradation and a safety parameter because it is considered pro-atherosclerotic and associated with increased risk of major adverse cardiovascular events in adults [19].

## Methods

This is a randomized parallel-group comparison in 32 preterm infants, carried out at Tübingen University Hospital, Germany. Infants were recruited from June 2022 to February 2024. The Institutional Review Board (project number 322/2019BO1) approved the protocol, and written informed consent was obtained prior to enrolment.

*Inclusion criteria*: gestational age at birth 28.0–32.0 weeks and enteral intake ≥ 150 ml/kg/d.

*Exclusion criteria*: acute illness, diseases concerning the lipid metabolism especially of the gastrointestinal tract (enterostomy, pancreatic insufficiency, cholestasis), systemic therapy with corticosteroids, renal insufficiency (creatinine > 1.5 mg/dl), significant left- or right ventricular failure with liver congestion, or missing consent.

*Study procedure*: Using sealed opaque envelopes, infants were randomly allocated to the 4 different choline compounds as well as to one of the two blood sampling intervals: 3 h or 6 h after first and last dose.

*Supplementation*: 30 mg/kg/d ‘choline equivalent’ (i.e., 0.288 mmol/kg/d of choline) were given for 48 h as: choline chloride (40 mg/kg/d), choline bitartrate (72 mg/kg/d), GPC (73,5 mg/kg/d), or egg-PC (217 mg/kg/d).

Choline chloride (CAS: 67-48-1), choline bitartrate (CAS:87-67-2) and α-glycerophosphocholine (GPC; CAS: 28319-77-9) have GRAS (Generally Recognized As Safe) status, of which GPC is one of the major natural choline compounds in human milk. Egg-phosphatidylcholine (egg-PC; CAS: 8002-43-5) is a natural choline source in human nutrition and PC is a natural component of human milk as well. Supplements were obtained from Lipoid (Ludwigshafen, Germany; GPC, egg-PC) and Merck (Darmstadt, Germany; choline chloride, choline bitartrate). Aliquots of dry substances were transferred to sterile plastic vials by Rainfarn-Apotheke (RAINFARN Gesundheit, Rainfarnstr. 36, 80935 München) and stored at 4 °C (choline chloride, choline bitartrate, and GPC) or − 18 °C (egg-PC) until use. Before use, aliquots were dissolved in 5 ml room-tempered sterile water on the day of use and stored at 4 °C between feeds for a maximum of 24 h. Thereafter fresh aliquots were prepared. For egg-PC, the container was well shaken after addition of water to achieve a homogenous, milky-white emulsion. 2 ml/kg/d equaling 30 mg/kg/d choline equivalent were administered in 6–8 fractions with the daily milk feeds.

Infants received fortified milk from their own mother, fortified human donor milk or preterm infant formula as detailed in Table 1. Based on previous analyses, breast milk provides ~ 21 mg/kg/d of choline equivalent (thereof ~ 1.6 mg/kg/d of choline equivalent as PC), fortifier provides about 10 mg/kg/d of choline equivalent (thereof ~ 0.4 mg/kg/d as PC), and formula provides ~ 18 mg/kg/d of choline equivalent with very little choline from PC [7].

**Table 1:** Demographic data

		Total	Cholin chloride	Choline bitartrate	GPC	Egg-PC
Participants		32	8	8	8	8
Male/female		16/16	4/4	5/3	5/3	2/6
GA at birth (w)		31.1 (30.1–31.6)	31.3 (29.9–31.6)	31.6 (31.3–31.9)	30.7 (30.2–31.9)	30.8 (30.0–31.3.0.3)
PNA at first supplement dose (d)		14 (13–16)	14 (14–14)	15 (13–18)	15 (13–15)	14 (13–16)
Weight at birth (g)		1393 (1255–1483)	1405 (1248–1693)	1440 (1408–1536)	1403 (1353–1473)	1115 (965–1313)
Weight at first dose of supplement (g)		1587 (1444–1761)	1653 (1506–1833)	1673 (1613–1735)	1576 (1434–1603)	1380 (1213–1666)
Nutrition at supplement start	Fortified breast milk	11	1	4	5	1
	Fortified breast milk + formula	21	7	4	3	7
	Formula	0	0	0	0	0

Data are shown as median (Quartile 1–Quartile 3). Every infant receiving breast milk or human donor milk, received FM85% (cow milk-based multicomponent human milk fortifier, Nestle, Frankfurt, Germany) with 4 g/100 ml in each meal (~ 11 mg choline in 150 ml [7]). The formula was Beba for preterms < 1800 g (~ 25 mg choline/150 ml [7]) (Nestle, Frankfurt, Germany)

*Of note*: the infants in the egg-PC group more commonly were female and tended to be lighter than the infants receiving the other supplements

Abbreviations: GA, gestational age; PNA, postnatal age; d, days; w, weeks

Three EDTA-blood samples of 0.2 ml each were taken: for baseline at < 72 h before start of supplementation, and, as randomly assigned, at either 3 h or 6 h after first and last dose (i.e., at 3 h and 51 h or at 6 h and 54 h after first dose, where first dose = 0 h and last dose = 48 h). These timepoints were chosen because changes in plasma concentrations were expected to become evident at 3–6 h, and a near-equilibrium state was expected at 48–51 h, which is beyond 10 half-lives (estimated at < 3 h) for plasma choline [18]. Samples were taken together with clinically indicated blood tests to avoid additional needle pricks. Blood was immediately centrifuged at 1000 x *g* at room temperature for 10 min and plasma was separated and stored at − 80 ℃ until analysis. Samples were analyzed in four batches, and all samples from an infant were measured in the same sample preparation set.

*Chemical analysis*: Samples were prepared and analyzed as previously described. Blood plasma (50 µL) was extracted according to Bligh and Dyer [20] and diarachidoyl-PC (PC20:0/20:0) and D_4_-choline chloride were used as internal standards. After centrifugation, the lower chloroform and the upper water: methanol phase were stored at − 80 ℃ and analyzed for PC molecular species, and for choline and its water-soluble metabolites, respectively, using LC-H-ESI-MS/MS as described before [17]. Dimethylglycine (DMG), methionine and carnitine were analyzed using the same extracts and method, with mass by charge (m/z) transitions of + 104.0→+58.1 for DMG, + 150.1→+61.05 for methionine and + 162.0→60.1 for carnitine. The coefficients of variation by compound indicated as % of the mean were: choline (5.1%), betaine (5.7%), dimethylglycine (12.4%), TMAO (11.5%), carnitine (7.9%), methionine (9.6%), PC (5.4%), SPH (3.8%), and lyso-PC (3.5%). For TMAO, the lower limit of detection was 10nmol/l, with 23 of the 94 samples below this limit.

*Statistics*: Calculation of sample size was based on plasma choline levels of preterm infants with and without choline supplementation from previous studies. Plasma free choline levels were calculated from means and standard deviations from our previous choline supplementation trial [16] (18.4 ± 4.8 µmol/L without and 37.7 ± 11.0 µmol/L with supplemental 30 mg/kg/d choline equivalent corresponding to 40 mg/kg/d choline chloride for 10 days). Assuming normal distribution of the data, a similar effect size and α ≤ 0.05, five individuals in each group (4 supplements) had to be analyzed to verify this clinically relevant impact on choline concentration with a power of 80%. Since these data were only a rough estimate of potentially relevant differences for power analysis, we enrolled 8 infants for each supplement group (total of 32). The primary outcome was defined as the area under the concentration curve (AUC) of the choline plasma concentration at 3–51 h and 6–54 h. AUCs were calculated for each infant based on the trapezoidal rule. Secondary outcome variables were the plasma concentrations of choline and metabolites at 3, 6, 51 and 54 h compared to 0 h. Post hoc, the sum of choline and betaine plasma concentrations were also analyzed, as up to 40% of choline is metabolized to betaine [21]. This combined measurement may be a more valid parameter of choline status [22]. Furthermore, DMG was added as a demethylation product of betaine [5].

Statistical and graphical analyses were done using Excel 2010 (Microsoft Corporation, USA) and GraphPad Prism Version 8.4.0 (GraphPad Software, San Diego, California, USA). Normal distribution was tested with the Shapiro Wilk test. Because several parameters were not normally distributed, results are shown as medians with 25th and 75th percentiles and non-parametric tests were used (Kruskall Wallis test). A significance level of *p* = 0.05 was applied. We did not correct for multiple testing in this exploratory analysis.

## Results

**Fig. 1:**
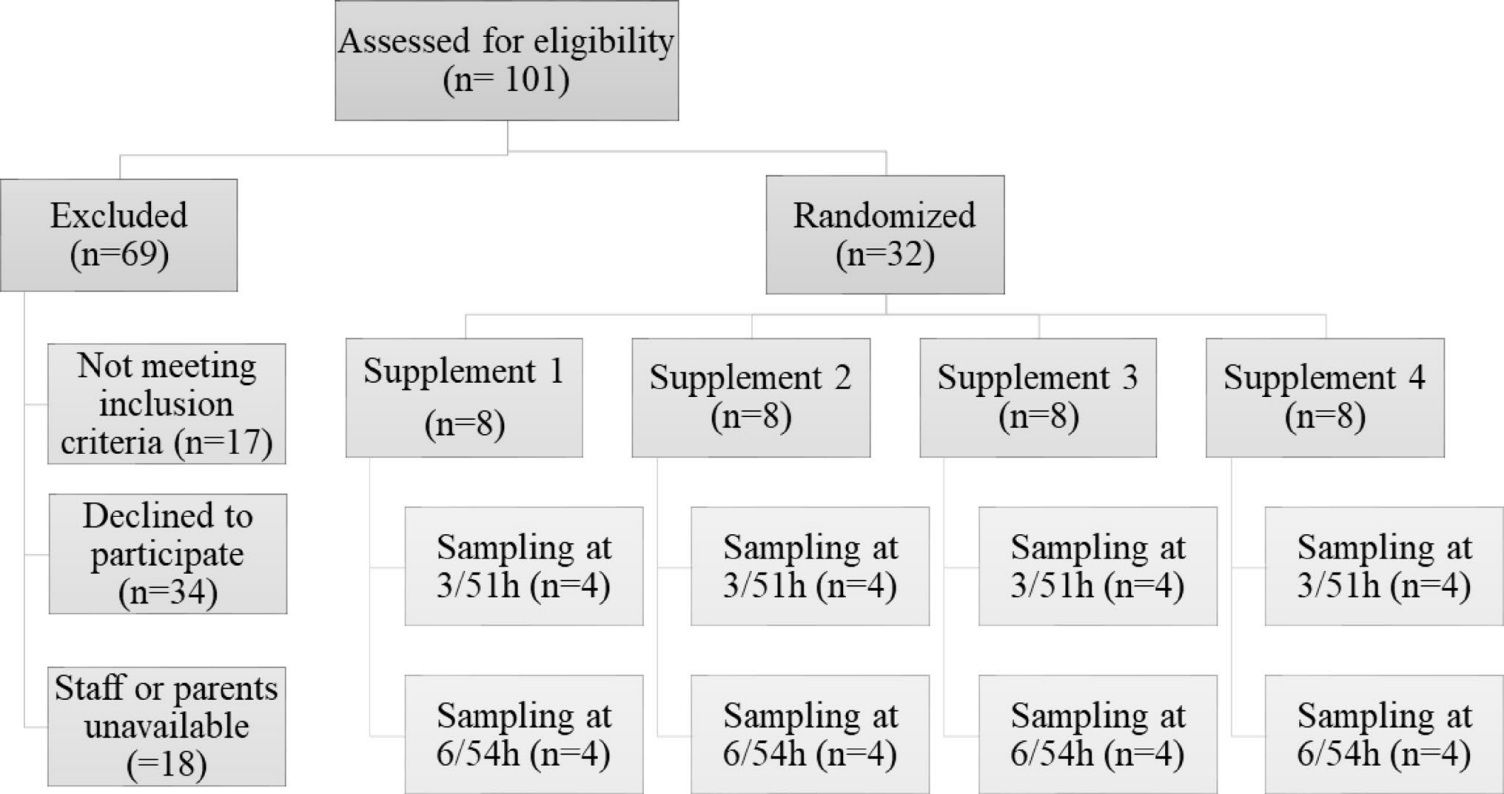
Participant flow. Participant Flow of 32 infants recruited into the trial and randomly assigned to 1 of 4 choline supplements (Glycerophosphocholine, Choline Chloride, Choline Bitartrate, and Egg-Phosphatidylcholine). 54 h-blood samples were not collected for 1 infant allocated to glycerophosphocholine and 1 infant assigned to choline chloride because their parents wished to discontinue the study

Participant flow and demographics are shown in Fig. 1; Table 1. Two infants did not complete the study: the twins ID 12 (choline chloride, blood sample at 6 h) and ID 13 (GPC, blood sample at 6 h) had only two blood samples taken and the supplementation was stopped after 8 h, because one of these twins had acutely increased apnea and parents withdrew study consent for both infants. This twin was treated with Piperacillin/Tazobactam for suspected late-onset bacterial infection and all symptoms resolved within 36 h.

### Concentrations of choline and choline related parameters at baseline

At baseline, median (Quartile 1–Quartile 3) choline plasma concentrations were similar in all groups (*p* = 0.2) (Table 2), and was 23.4 (19.8–26.1) µmol/L for all infants in the study (Table 3). Similarly, there were no differences in median plasma concentrations for betaine, GPC, TMAO or PC at 0 h, ranging from 29.4–40.2 µmol/L, 1.04–1.32 µmol/L, 0.03–0.08 µmol/L and 1.57–1.70 mmol/L, respectively (Table 2). Notably, plasma choline and betaine concentrations were highly variable, with 25% of values below 19.8 µmol/l for choline and below 25.4 µmol/L for betaine.

**Table 2:** Plasma concentrations of choline and its metabolites before, during and after choline supplementation by individual choline compounds

Supplement →	Choline chloride	Choline bitartrate	GPC	Egg-PC
*Plasma choline (µmol/L)*				
0 h	21.4 (17.9–24.3)	24.3 (22.4–25.2)	26.8 (24.5–28.9)	21.1 (16.6–23.6)
3 h	23.2 (21.9–23.2)	21.1 (20.0–23.7.0.7)	25.4 (22.6–28.6)	20.2 (18.3–22.6)
6 h	22.7 (17.5–25.8)	25.1(24.0–27.2)	38.2 (31.8–46.1)*^,#^	22.6 (17.7–26.7)
51 h	35.6 (32.6–36.1)	35.1(32.8–36.3)**	36.0 (32.9–38.5)*	32.0 (26.6–37.0)
54 h	28.8 (23.4–42.2)	31.2 (29.6–32.9)	32.1 (27.7–33.2)	27.9 (24.2–32.7)
*Plasma betaine (µmol/L)*				
0 h	40.2 (32.2–48.3)	36.3 (24.3–44.4)	35.2 (31.3–44.1)	29.4 (22.2–34.7)
3 h	43.9 (39.6–46.4)	35.6 (32.9–40.3)	39.2 (35.9–43.2)	29.9 (26.0–41.6.0.6)
6 h	37.9 (33.0–39.7)	29.2 (26.4–32.3)	37.6 (37.1–44.2)	29.5 (24.1–32.3)
51 h	68.7 (66.2–81.5)*	57.1 (53.1–64.6)*	58.9 (52.0–66.7.0.7)*	46.1 (37.8–62.5)
54 h	51.0 (45.1–64.5)	50.8 (46.9–55.6)	38.7 (38.0–41.0)	48.8 (38.3–59.7)
*Plasma combined choline + Betaine (µmol/L)*				
0 h	59.7 (54.7–72.1)	61.1 (46.3–70.1)	66.1 (57.9–68.6)	50.5 (37.1–62.2)
3 h	67.0 (62.8–68.3)	60.0 (53.4–66.8)	63.7 (62.0–67.3.0.3)	49.1 (43.3–64.2)
6 h	62.4 (50.5–63.7)	55.6 (54.0–57.3.0.3)	75.8 (68.9–90.2)*^,#^	52.1 (45.2–55.5)
51 h	104.3 (102.3–114.0)*	89.6 (83.3–101.0)*	96.2 (91.9–99.6)**	78.1 (64.4–99.4)
54 h	79.2 (67.9–106.7.9.7)	79.1 (77.9–84.1)	70.8 (65.7–74.1)	88.5 (79.8–92.6)
*Plasma GPC (µmol/L)*				
0 h	1.23 (0.96–1.31	1.32 (1.11–1.47)	1.04 (0.93–1.30)	1.12 (0.87–1.22)
3 h	0.79 (0.78–0.86)	1.08 (0.89–1.19)	1.12 (1.06–1.14)	1.03 (0.95–1.15)
6 h	0.75 (0.72–0.96)	1.30 (1.12–1.44)	1.24 (0.88–1.60)	1.07 (0.78–1.33)
51 h	1.02 (0.98–1.07)	1.15 (1.08–1.22)	0.94 (0.78–1.10)	0.88 (0.84–0.88)
54 h	0.85 (0.80–0.94)	0.78 (0.69–0.88)	1.16 (1.11–3.64)	0.73 (0.65–0.97)
*Plasma TMAO (µmol/L)* ^§^				
0 h	0.08 (0.00–0.17.00.17)	0.08 (0.00–0.10.00.10)	0.14 (0.1–0.18)	0.03 (0.00–0.11.00.11)
3 h	0.05 (0.02–0.08)	0.06 (0.05–0.08)	0.16 (0.14–0.20)	0.06 (0.00–0.12)
6 h	0.21 (0.20–0.28)	0.14 (0.09–0.17)	0.20 (0.11–0.42)	0.02 (0.00–0.04)
51 h	0.08 (0.06–0.09)	0.11 (0.10–0.20)	0.13 (0.11–0.65)	0.11 (0.05–0.21)
54 h	0.21 (0.15–0.35)	0.12 (0.09–0.37)	0.10 (0.09–0.12)	0.00 (0.00–0.01)
Total PC (mmol/L)				
0 h	1.70 (1.56–1.82)	1.56 (1.49–1.64)	1.63 (1.43–1.77)	1.58 (1.54–1.77)
3 h	1.49 (1.43–1.59)	1.55 (1.45–1.70)	1.60 (1.59–1.69)	1.53 (1.45–1.62)
6 h	1.72 (1.68–1.83)	1.59 (1.55–1.74)	1.87 (1.71–1.99)	1.89 (1.73–2.02)
51 h	1.55 (1.47–1.62)	1.70 (1.55–1.87)	1.62 (1.56–1.70)	1.58 (1.51–163)
54 h	1.81 (1.66–1.94)	1.55 (1.48–1.70)	1.78 (1.66–1.97)	1.97 (1.78–2.12)

Data are shown as medians (Quartile 1–Quartile 3). For actual numbers of plasma samples in each cell please refer to Fig. 1. Abbreviations: GPC, alpha-glycerophosphocholine; h, hours; PC, phosphatidylcholine; TMAO, Trimethylamine oxide;

There were no statistically significant between group differences at any timepoint except for combined concentrations of choline and betaine at 6 h

^#^, *p* < 0.05 versus all other supplements; *, *p* < 0.05 versus 0 h; **,* p * < 0.01 versus 0 h

^§^, For TMAO, the lower limit of detection was 10nmol/l, with 23 of the 94 samples below this limit – these are indicated as “0.00 µmol/L”

**Table 3:** Plasma concentrations of choline and its metabolites before, during and after choline supplementation

	0 h (*n* = 32)	3 h (*n* = 16)	6 h (*n* = 16)	51 h (*n* = 16)	54 h (*n* = 14)
Choline (µmol/L)	23.4 (19.8–26.1)	22.0 (20.0–25.1.0.1)	25.9 (22.7–31.0)	34.2*** (30.2–37.0)	30.9** (24.6–56.0)
Betaine (µmol/L)	34.2 (25.4–44.0)	36.5 (32.8–45.2)	35.0 (28.4–37.9)	59.0*** (51.1–69.6)	46.9* (40.3–56.0)
Choline + Betaine (µmol/L)	59.7 (46.3–68.9)	63.1 (53.7–67.4)	59.6 (51.9–64.8)	95.0*** (84.6–102.3.6.3)	77.5** (70.1–88.7)
GPC (µmol/L)	1.2 (1.0–1.4.0.4)	1.0 (0.9–1.2)	1.0 (0.8–1.4)	0.9 (0.9–1.1)	0.8* (0.8–1.0.8.0)
TMAO (µmol/L)	0.09 (0.00–0.14.00.14	0.10 (0.02–0.12)	0.12 (0.07–0.20)	0.11 (0.08–0.15)	0.11 (0.01–0.14)
DMG (µmol/L)	9.2 (7.1–12.5)	9.3 (6.6–12.3)	8.3 (7.5–12.1)	13.3* (8.7–18.4)	11.7* (10.2–18.3)
Methionine (µmol/L)	4.7 (3.9–5.8)	4.5 (4.2–5.2)	4.7 (4.1–5.7)	4.7 (4.1–6.4)	5.4 (4.9–6.0.9.0)
Carnitine (µmol/L)	22.3 (16.8–24.7)	21.3 (18.8–23.4)	25.3 (20.4–27.4)	20.3 (15.7–22.4)	25.3 (22.7–27.4)
PC (mmol/L)	1.59 (1.48–1.77)	1.59 (1.46–1.67)	1.74 (1.61–1.98)	1.58 (1.52–1.67)	1.78 (1.58–1.98)

Data are medians (Quartile 1–Quartile 3) over all infants together. Abbreviations: n, number of infants; h, hours; GPC, alpha-glycerophosphocholine; PC, phosphatidylcholine; TMAO, trimethylamine oxide; DMG, dimethylglycine; *, *p* < 0.05 versus 0 h; **, *p* < 0.01 vsersus 0 h; ***, *p* < 0.001 versus 0 h

### Effects of different choline compounds on plasma concentrations and areas under the concentration curves (AUC)

AUCs for choline plasma concentrations did not differ significantly between the four choline supplements (Table 4). There was a trend towards a higher median AUC for choline with GPC compared to the other components (*p* = 0.069). After 48 h of supplementation, choline plasma concentrations had increased to a similar extent following egg-PC supplementation as following the water-soluble choline compounds. All choline supplements similarly increased betaine concentrations and betaine AUCs (Table 4), as well as combined choline and betaine concentrations. However, GPC showed higher values at 6 h compared to other supplements (Table 2). There were no statistically significant differences between supplements for DMG, and no effects on methionine and carnitine concentrations.

**Table 4:** Areas under the concentration curves and mean plasma concentrations of choline and betaine during and after choline supplementation by individual choline compounds

	Choline chloride	Choline bitartrate	GPC	Egg-PC
Choline AUC^**a**^ (µmol/L*h)	1234 (1145–1423)	1385 (1311–1482)	1611 (1366–1628)	1252 (945–1447)
Choline mean concentration^**b**^ (µmol/L)	23.6 (21.5–27.9)	27.2 (24.6–27.5)	29.8 (26.1–31.9)	24.6 (17.5–26.8)
Betaine AUC^**a**^ (µmol/L*h)	2373 (2095–3068)	2126 (1993–2170)	2048 (1887–2355)	1901 (1457–2113
Betaine mean concentration^**b**^ (µmol/L)	46.5 (38.8–60.2)	40.0 (38.3–41.9)	40.2 (34.9–46.2)	35.2 (28.6–40.8)

Areas under the concentration curves and mean plasma concentrations of choline and betaine during and after choline supplementation are shown as median (Quartile 1–Quartile 3) by individual choline compounds. ^**a**^AUCs of plasma choline and betaine concentrations (herein combined for 3–51 h and 6–54 h) were calculated with the trapezoidal rule. ^**b**^Mean concentrations were interpolated from individual concentration at 3 h and 51 h as well as at 6 h and 54 h, respectively. (Without ID 12 and 13, as no values beyond 6 h were available)

### Time course of concentration changes in response to choline supplementation

Evaluating plasma concentration changes in all participants together (irrespective of supplemented choline compound), there were no significant increases in plasma concentrations of choline, betaine, the sum of both or DMG at 3 h or at 6 h after start of supplementation. However, concentrations were increased by + 46%, + 73%, + 59% and + 44%, respectively, at 51 h, and by + 32%, + 37%, + 30%, and + 27%, respectively, at 54 h (Table 3). GPC resulted in an increase in choline plasma concentration at 6 h compared to baseline (*p* = 0.01), whereas the other compounds did not (Table 2, Supplemental Figs. S1 and S2). Methionine and carnitine levels did not change over the study period (Table 3, Supplemental Fig.  S2).

### TMAO

After the administration of all choline supplements, except egg-PC, we observed a small increase in plasma TMAO concentration. However, there was considerable inter-subject variability, and all TMAO concentrations were less than 1 µmol/l (Table 2 and Supplemental Fig. S1).

### Phosphatidylcholine (PC)

Plasma PC concentrations did not change following choline supplementation, neither for any individual supplement nor for all infants together (Tables 2 and 4). This also applied to PC subclasses. Notably, even after > 48 h, choline supplementation with egg-PC did not change the molecular composition of PC or the fractions of PC subclasses comprising mono- or poly-unsaturated fatty acids (oleoyl-PC, linoleoyl-PC, ARA-PC and DHA-PC) (Supplemental Fig. S3).

## Discussion

Supplementation of preterm infants with additional 30 mg/kg/d choline equivalent provided by four different choline compounds increased plasma concentrations of choline and betaine to a similar degree (about 30–50% above baseline after 48 h of supplementation). Considering the kinetics of this increase, a single supplemental dose of 3.75 mg/kg or 5.0 mg/kg choline equivalent (3 h blood sample) or two repeated doses (6 h blood sample) were not sufficient to increase plasma choline concentrations compared to baseline for all compounds, except for GPC, which already showed an increase after two doses (at 6 h). For all compounds, choline plasma concentrations tended to decrease at 6 h compared to 3 h after end of supplementation.

These data contrast with results from previous studies with single-dose Deuterium-labelled (D9-) choline compounds in preterm infants [23] or overnight-fasting adults [18], or a single daily-adequate-intake dose (550 mg/kg choline equivalent) in overnight-fasting adults [17]. In those studies, peak levels were measured at 1.5–2.5 h (except for PC), followed by a rapid clearance of choline from plasma. However, those studies were in-vivo pulse-chase experiments to analyze metabolism in contrast to this study administering multiple small doses to achieve equilibrium data.

The cumulative daily dose of 30 mg/kg/d choline equivalent was chosen based on the assumption and previous data [16] that supplemental 30 mg/kg/d of choline in addition to the ‘standard’ choline supply of about 20–30 mg/kg/d is required to achieve plasma concentrations near-equivalent to a fetus in utero at 28 to 32 wk postmenstrual age. At this developmental stage in utero, fetal plasma choline concentrations are 47 (41–62) µmol/L [5], compared to the 23 (20–26) µmol/L observed in the participants in this study at baseline. However, after 48 h of supplementation, choline plasma concentrations were ‘only’ about 30–50% higher than at baseline (34 (30–37) µmol/l), and still below corresponding fetal concentrations for all compounds. The comparison with our previous study, supplementing the same dose of choline for 10 d and reaching near-fetal choline concentrations [16], indicates that it may take more than 48 h of multi-dose supplementation to reach an equilibrium. The observed decrease in plasma concentrations from 51 h to 54 h, sampled 3 h and 6 h after of the last dose of choline, may indicate that plasma choline levels rapidly decrease upon cessation of supply, as was previously shown for adults [15, 17] and corresponding to a high turnover.

In our previous studies, we demonstrated that the kinetics of absorption and subsequent metabolism of PC is fundamentally different from that of water-soluble choline compounds [17, 18]. PC is absorbed as lyso-PC after phospholipase cleavage and, thereafter, to a greater extent re-acylated with PUFAs such as linoleic acid, ARA and DHA [13] and to a lesser extent further degraded to free choline if compared to the water-soluble compounds [17]. However, this study showed that 48 h of multi-dose egg-PC supplementation increased free plasma choline to a similar extent as the water-soluble compounds and exerted no significant effect on plasma PC concentration or composition.

Due to the high content in linoleic acid in human milk and preterm infant formula, the lipidome of preterm infants is distorted with higher contents of linoleic acid and diminished content of ARA and DHA in plasma PC, erythrocyte membrane PC and subcutaneous fat triglycerides when compared to term newborns at birth [24], indicating that intrauterine nutrition via the placenta, which enriches ARA and DHA in the fetus, is neither fully substituted by human milk nor by current formula. Based on our own unpublished measurements, the daily supplemental dose of egg-PC (218 mg/kg/d PC or 30 mg/kg/d choline equivalent) provided 35.1, 29.5, 8.5, and 5.1 mg/kg/d of additional oleic acid, linoleic acid, ARA and DHA. Compared to a total intake of 2000, 700, 45 and 25 mg/kg/d of oleic acid, linoleic acid, ARA and DHA with breast milk [7], a change in plasma lipid profile by egg-PC supplementation could not be expected.

We previously demonstrated that co-supplementation of choline chloride and DHA increased DHA-PC compared to exclusive DHA supplementation [16] and D9-labelled 1-palmitoyl-2-oleoyl-PC increased LC-PUFA-D9-PC more than D9-labelled water-soluble choline compounds [23]. It remains to be investigated if a prolonged co-administration of (egg-)PC together with additional ARA and DHA will achieve a more physiological pattern of PC subgroups, i.e., decreased linoleic acid-containing PC and higher fractions of ARA-PC and DHA-PC. Supplementation of ARA and DHA in a ratio of 2:1 seemed to reduce the rate of retinopathy of prematurity [25] and might also have positive effects on respiratory outcomes [26]. Because both ARA and DHA are predominantly found in the PC fraction of plasma lipoproteins [2], co-supplementation of PC with ARA and DHA may further improve the distribution of these essential PUFAs to the infants’ lung, brain, eye and other organs and enhance beneficial effects.

No relevant side effects were observed. Therefore, all tested supplements appear to be safe in preterm infants. Two infants (Twins: ID 12, choline chloride and ID 13, GPC) showed increased frequency of apnea early after onset of supplementation, and the study intervention was stopped at the request of the infants’ parents. However, these symptoms were interpreted as an unremarkable fluctuation in the clinical condition, frequently observed in preterm infants around the age of 14 days [27]. One of these infants had suspected late-onset infection and was treated with Piperacillin and Tazobactam. We do not see a causal relationship to the intake of choline.

Supplementation with choline chloride, choline bitartrate or GPC, but not with (egg-)PC or phosphorylcholine, resulted in increased TMAO in adults [17, 18]. TMAO is the oxidation product of TMA (a bacterial degradation product of choline and other trimethyl ammonium compounds) and is considered a cardiovascular risk factor [28]. In adults, we observed the highest increase in TMAO after the intake of choline bitartrate [17]. In healthy adults, TMAO concentrations of ~ 3 µmol/l were measured following a single dose of 550 mg choline equivalent [17], but values can increase to > 10µmol/l in patients with intestinal bacterial colonization [15]. By contrast, all premature infants in this study had values < 1 µmol/l, confirming previous data [16]. While TMAO formation was low or absent, this does not exclude bacterial degradation of choline to trimethylamine (TMA), as the oxidation of TMA to TMAO requires the hepatic flavin-containing monooxygenase 3 (FMO3; EC 1.14.13.148) the expression of which only develops postnatally [29] and, hence, may be insufficient in preterm infants. If TMAO is the relevant mediator for cardiovascular events (and not the volatile TMA), our findings seem to indicate that choline supplementation in preterm infants does not evoke a pro-thrombotic/cardiovascular risk.

Choline salts, mainly of chloride and bitartrate, are frequently used in formula, milk fortifiers for preterm and term infants as well as food supplements [7], whereas organic choline esters (GPC, phosphoryl-choline and PC) are present in human milk. Two major commercially available choline compounds naturally present in human milk are the water-soluble GPC and the phospholipid PC [6, 7, 30]. In this study, we used egg-PC as a lipidic choline source, as it is rich in oleic acid and contains some amount of arachidonic (ARA) and docosahexaenoic acid (DHA). Plant-derived PC, by contrast, is devoid of ARA and DHA, and contains large amounts of linoleic acid (LA) [31]. Phosphorylcholine as the second major choline compound in breast milk [6, 7], is more rapidly absorbed and does not seem to be transformed to TMA in adults [18], however it is momentarily not available on the market in suitable quality and quantity for supplementation studies.

### Limitations

This study is limited by the small numbers of plasma samples per individual infant. While investigating the kinetics of choline compounds in adult healthy volunteers enables sequential blood drawing in 30 min intervals, this is not feasible in preterm infants for ethical reasons. Hence, selecting time points like 3 h and 6 h after start and end of supplementation was derived from adult data, previous studies in infants, and the assumption of a faster choline turnover in infants compared to adults [23, 32]. Additionally, our sample size calculation was based on a comparison of plasma concentrations between supplemented and non-supplemented preterm infants (in the absence of better data), whereas here, we studied AUC differences between groups supplemented with identical doses of “choline equivalent” administered through different choline compounds. Therefore, retrospectively, this study was underpowered to evaluate the actual observed differences in the AUC of plasma levels. Nevertheless, this study demonstrates for the first time that 48 h of PC-supplementation in preterm infants increases choline plasma levels to a similar degree as supplementation of the water-soluble choline compounds. Finally, all participants were stable preterm infants. While this is justified by the explorative and non-curative nature of our study, absorption kinetics and intra-intestinal catabolism of choline components may be different in infants after intestinal resection, with pancreatic insufficiency or small intestinal bacterial colonization. However, this will have to be investigated under different study settings.

## Conclusion

Choline chloride, choline bitartrate, GPC and PC all increased plasma choline levels to a similar extent upon continuous administration for 48 h in very preterm infants, with GPC leading to a faster increase already evident after 6 h. PC, which is metabolized differently than the other choline compounds and is remodeled in the intestine to LC-PUFA-PC, similarly increased plasma choline values after 48 h.

## Supplementary Information

Below is the link to the electronic supplementary material.

Supplementary Material 1

## Abbreviations

Ach: Acetylcholine
AI: Adequate intake
ARA: Arachidonic acid
AUC: Area under the concentration curve
D: Deuterium
d: Day(s)
DHA: Docosahexaenoic acid
EDTA: Ethylenediaminetetraacetate
EFSA: European food safety authority
EPA: Eicosapentaenoic acid
ESPGHAN: European Society of the Paediatric Gastroenterology, Hepatology, and Nutrition
GPC: Alpha-glycerophosphorylcholine
h: Hours
LC-PUFA: Long-chain poly-unsaturated fatty acid
PC: Phosphatidylcholine
SPH: Sphingomyelin
TMA: Trimethylamine
TMAO: Trimethylamine oxide

## References

[1] Bernhard W (2016) Lung surfactant: function and composition in the context of development and respiratory physiology. Ann Anat 208:146–150 27693601 10.1016/j.aanat.2016.08.003

[2] Bernhard W, Maas C, Shunova A et al (2018) Transport of long-chain polyunsaturated fatty acids in preterm infant plasma is dominated by phosphatidylcholine. Eur J Nutr 57:2105–2112 28638995 10.1007/s00394-017-1484-1

[3] Zeisel SH (2006) Choline: critical role during fetal development and dietary requirements in adults. Annu Rev Nutr 26:229–250 16848706 10.1146/annurev.nutr.26.061505.111156 PMC2441939

[4] Arumugam MK, Paal MC, Donohue TM Jr. et al (2021) Beneficial effects of betaine: a comprehensive review. Biology (Basel). 10.3390/biology10060456 34067313 10.3390/biology10060456 PMC8224793

[5] Bernhard W, Raith M, Kunze R et al (2015) Choline concentrations are lower in postnatal plasma of preterm infants than in cord plasma. Eur J Nutr 54:733–741 25148882 10.1007/s00394-014-0751-7

[6] Maas C, Franz AR, Shunova A et al (2017) Choline and polyunsaturated fatty acids in preterm infants’ maternal milk. Eur J Nutr 56:1733–1742 27164830 10.1007/s00394-016-1220-2

[7] Shunova A, Bockmann KA, Minarski M et al (2020) Choline content of term and preterm infant formulae compared to expressed breast milk-how do we justify the discrepancies? Nutrients. 10.3390/nu12123815 33322176 10.3390/nu12123815 PMC7763895

[8] Bernhard W, Full A, Arand J et al (2013) Choline supply of preterm infants: assessment of dietary intake and pathophysiological considerations. Eur J Nutr 52:1269–1278 22961562 10.1007/s00394-012-0438-x

[9] Bernhard W, Poets CF, Franz AR (2019) Choline and choline-related nutrients in regular and preterm infant growth. Eur J Nutr 58:931–945 30298207 10.1007/s00394-018-1834-7

[10] Hollenbeck CB (2012) An introduction to the nutrition and metabolism of choline. Cent Nerv Syst Agents Med Chem 12:100–113 22483274 10.2174/187152412800792689

[11] Lockman PR, Allen DD (2002) The transport of choline. Drug Dev Ind Pharm 28:749–771 12236062 10.1081/ddc-120005622

[12] Li Z, Agellon LB, Vance DE (2007) Choline redistribution during adaptation to choline deprivation. J Biol Chem 282:10283–10289 17283071 10.1074/jbc.M611726200

[13] Bernhard W (2021) Choline in cystic fibrosis: relations to pancreas insufficiency, enterohepatic cycle, PEMT and intestinal microbiota. Eur J Nutr 60:1737–1759 32797252 10.1007/s00394-020-02358-2

[14] Bernhard W, Shunova A, Boriga J et al (2025) Low plasma choline, high trimethylamine oxide, and altered phosphatidylcholine subspecies are prevalent in cystic fibrosis patients with pancreatic insufficiency. Nutrients. 10.3390/nu17050868 40077735 10.3390/nu17050868 PMC11901616

[15] Bernhard W, Lange R, Graepler-Mainka U et al (2019) Choline supplementation in cystic fibrosis-the metabolic and clinical impact. Nutrients. 10.3390/nu11030656 30889905 10.3390/nu11030656 PMC6471815

[16] Bernhard W, Bockmann K, Maas C et al (2020) Combined choline and DHA supplementation: a randomized controlled trial. Eur J Nutr 59:729–739 30859363 10.1007/s00394-019-01940-7

[17] Bockmann KA, Franz AR, Minarski M et al (2022) Differential metabolism of choline supplements in adult volunteers. Eur J Nutr 61:219–230 34287673 10.1007/s00394-021-02637-6 PMC8783899

[18] Bockmann KA, Franz AR, Shunova A et al (2023) Different choline supplement metabolism in adults using deuterium labelling. Eur J Nutr 62:1795–1807 36840817 10.1007/s00394-023-03121-z PMC10195734

[19] Tang WH, Wang Z, Levison BS et al (2013) Intestinal microbial metabolism of phosphatidylcholine and cardiovascular risk. N Engl J Med 368:1575–1584 23614584 10.1056/NEJMoa1109400 PMC3701945

[20] Bligh EG, Dyer WJ (1959) A rapid method of total lipid extraction and purification. Can J Biochem Physiol 37:911–917 13671378 10.1139/o59-099

[21] Bernhard W, Raith M, Shunova A et al (2022) Choline kinetics in neonatal liver, brain and lung-lessons from a rodent model for neonatal care. Nutrients. 10.3390/nu14030720 35277079 10.3390/nu14030720 PMC8837973

[22] Minarski M, Maas C, Heinrich C et al (2023) Choline and betaine levels in plasma mirror choline intake in very preterm infants. Nutrients. 10.3390/nu15224758 38004152 10.3390/nu15224758 PMC10675502

[23] Bockmann KA, Bernhard W, Minarski M et al (2023) Choline supplementation for preterm infants: metabolism of four deuterium-labeled choline compounds. Eur J Nutr 62:1195–1205 36460779 10.1007/s00394-022-03059-8 PMC10030424

[24] Bockmann KA, Von Stumpff A, Bernhard W et al (2021) Fatty acid composition of adipose tissue at term indicates deficiency of arachidonic and docosahexaenoic acid and excessive linoleic acid supply in preterm infants. Eur J Nutr 60:861–872 32476053 10.1007/s00394-020-02293-2 PMC7900037

[25] Hellstrom A, Nilsson AK, Wackernagel D et al (2021) Effect of enteral lipid supplement on severe retinopathy of prematurity: a randomized clinical trial. JAMA Pediatr 175:359–367 33523106 10.1001/jamapediatrics.2020.5653 PMC7851754

[26] Wendel K, Aas MF, Gunnarsdottir G et al (2023) Effect of arachidonic and docosahexaenoic acid supplementation on respiratory outcomes and neonatal morbidities in preterm infants. Clin Nutr 42:22–28 36473425 10.1016/j.clnu.2022.11.012

[27] Poets CF, Roberts RS, Schmidt B et al (2015) Association between intermittent hypoxemia or bradycardia and late death or disability in extremely preterm infants. JAMA 314:595–603 26262797 10.1001/jama.2015.8841

[28] Canyelles M, Borras C, Rotllan N et al (2023) Gut microbiota-derived TMAO: a causal factor promoting atherosclerotic cardiovascular disease? Int J Mol Sci. 10.3390/ijms24031940 36768264 10.3390/ijms24031940 PMC9916030

[29] Al-Waiz M, Mikov M, Mitchell SC et al (1992) The exogenous origin of trimethylamine in the mouse. Metabolism 41:135–136 1736035 10.1016/0026-0495(92)90140-6

[30] Moukarzel S, Wiedeman AM, Soberanes LS et al (2019) Variability of water-soluble forms of choline concentrations in human milk during storage, after pasteurization, and among women. Nutrients. 10.3390/nu11123024 31835736 10.3390/nu11123024 PMC6949891

[31] Palacios LEW T (2005) Egg-yolk lipid fractionation and lecithin characterization. J Am Oil Chem Soc 82:571–578

[32] Goss KCW, Goss VM, Townsend JP et al (2020) Postnatal adaptations of phosphatidylcholine metabolism in extremely preterm infants: implications for choline and PUFA metabolism. Am J Clin Nutr 112:1438–1447 32778895 10.1093/ajcn/nqaa207 PMC7727469

