# Supplementary material for: Choline supplementation in preterm infants: effects of four different supplements on choline plasma concentrations
Source: Eur J Nutr. 2026 Jan 12;65(1):20. doi: 10.1007/s00394-025-03865-w (PMC12795929; doi:10.1007/s00394-025-03865-w)
Supplement: Supplementary file 1 — Supplementary Material 1 [file 394_2025_3865_MOESM1_ESM.docx]

Supplemental Figure S1: **Choline and Water-soluble Choline Metabolite Plasma Concentrations**


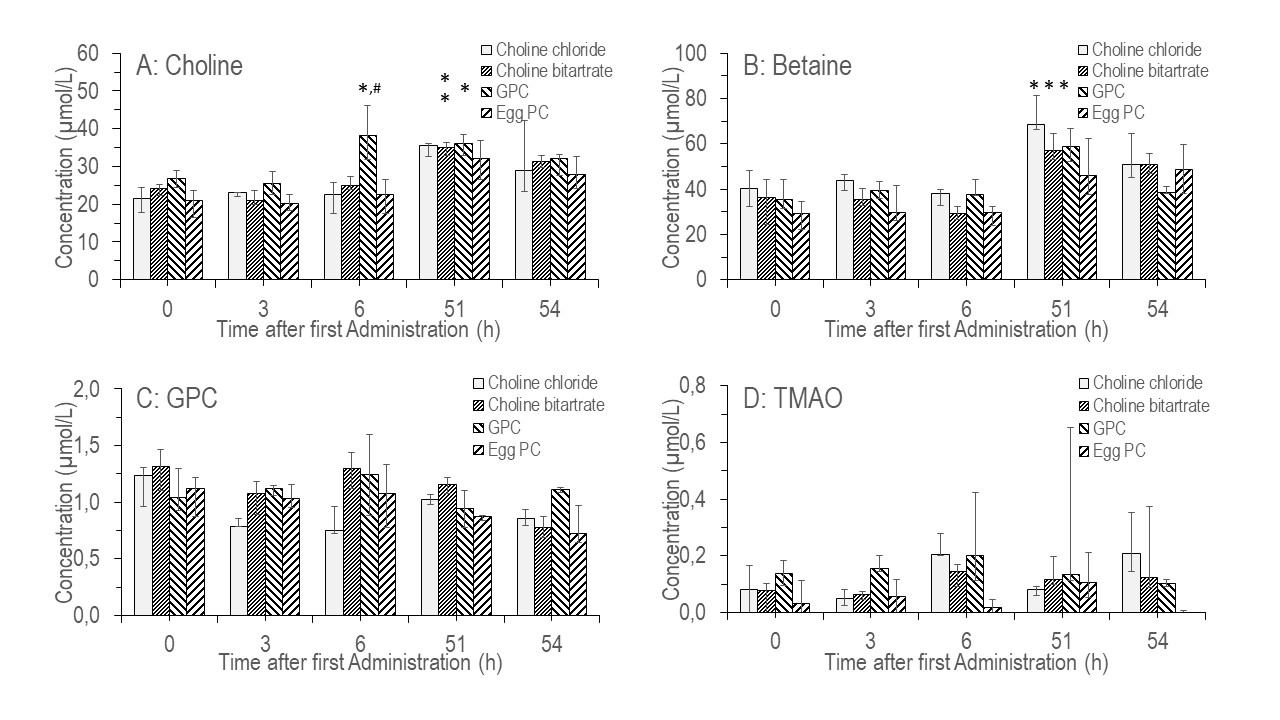


Choline and water-soluble choline metabolite concentrations in plasma by choline supplement and timing of blood samples (before start of supplementation (0h), as well as 3h, 6h, 51h and 54h after start of supplementation). Values are median (quartile 1 – quartile 3). Abbreviations: PC: phosphatidylcholine. GPC: glycerophosphocholine. TMAO: trimethylamineoxide. * p <0.05 vs. 0h; ** p<0.01 vs. 0h, # p<0.05 vs. all other supplements

Supplemental Figure S2: **Further** **Water-soluble Choline-Related Metabolite Plasma Concentrations**


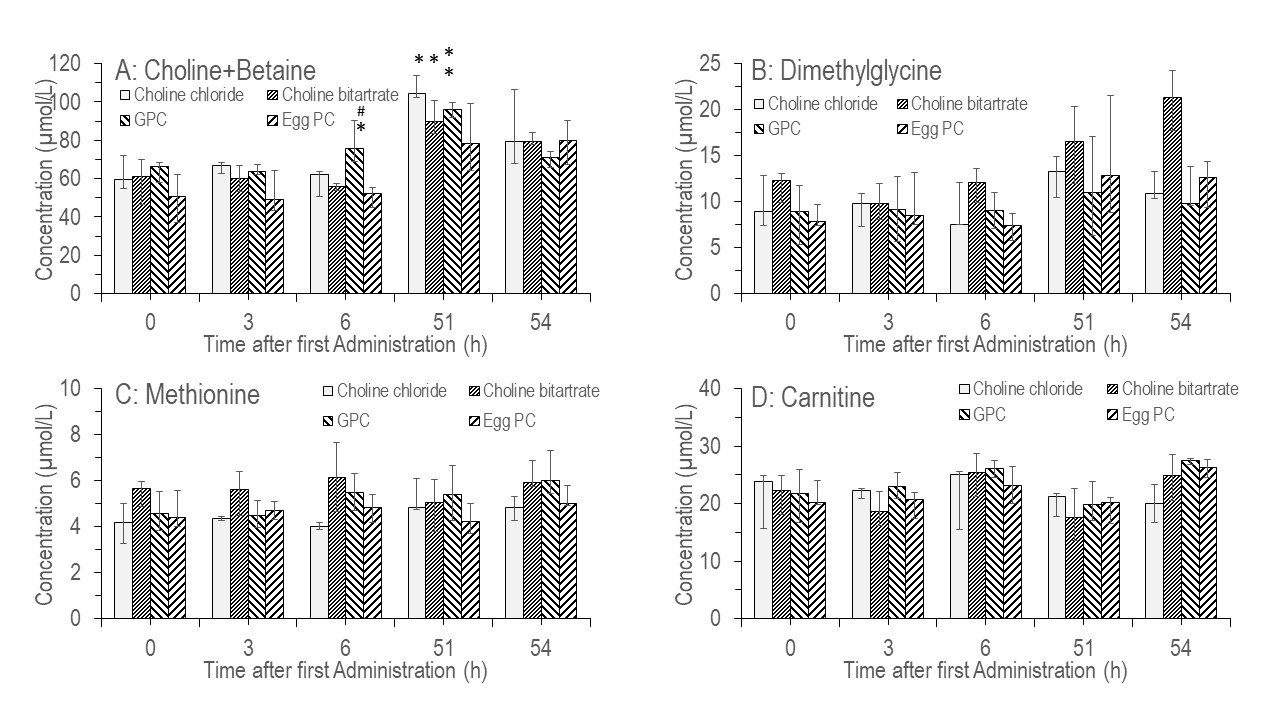


Further choline-related metabolite concentrations in plasma by choline supplement and timing of blood samples (before start of supplementation (0h), as well as 3h, 6h, 51h and 54h after start of supplementation). Data represent median (quartile 1 – quartile 3). Abbreviations: PC: phosphatidylcholine. GPC: glycerophosphocholine. * p <0.05 vs. 0h; ** p<0.01 vs. 0h, # p<0.05 vs. all other supplements

Supplemental Figure S3: **Molecular Composition of Plasma Phosphatidylcholine**


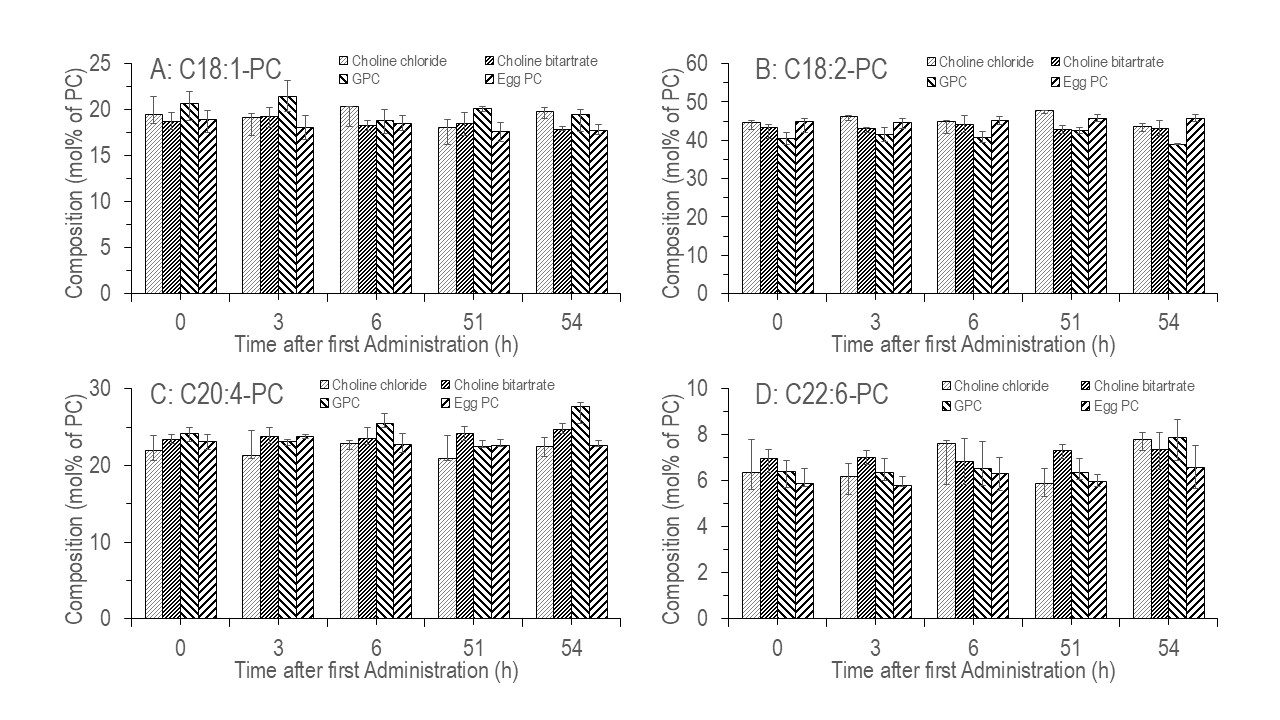


Fractions of major plasma phosphatidylcholine PC species, containing mono-unsaturated oleic acid (C18:1-PC), or poly-unsaturated linoleic acid (C18:2-PC), arachidonic acid (C20:4-PC) or docosahexaenoic acid (C22:6-PC) acid residue. Data are shown from start (0h) to 6h after end of supplementation (54h) across different supplement groups. Data represent median (quartile 1 – quartile 3). Abbreviations: PC: phosphatidylcholine. GPC: glycerophosphocholine
